# Accelerated Weight Gain Among Children During Summer Versus School Year and Related Racial/Ethnic Disparities: A Systematic Review

**DOI:** 10.5888/pcd11.130355

**Published:** 2014-06-12

**Authors:** Rebecca Franckle, Rachel Adler, Kirsten Davison

**Affiliations:** Author Affiliations: Rachel Adler, Kirsten Davison, Department of Nutrition, Harvard School of Public Health, Boston, Massachusetts.

## Abstract

**Introduction:**

The objective of this study was to compile and summarize research examining variations in weight gain among students during the summer in comparison to the school year, with a focus on racial/ethnic disparities and students who are at risk of overweight.

**Methods:**

A systematic search of PubMed and Embase was conducted. Reference lists of identified articles and Google Scholar were also reviewed. Studies that assessed summer weight gain in school children were included. Inclusion criteria were: 1) a focus on children and adolescents aged 5 to 17 attending school; 2) a measured body composition before and after the summer vacation; 3) English-language articles; and 4) publication in a peer-reviewed journal since January 1, 1990. Data were extracted from selected studies in the following categories: study purpose, setting, study design, population, sample size, data collection method, and findings.

**Results:**

Seven eligible studies were included in the review. Six of the 7 studies reported accelerated summer weight gain for at least a portion of the study population, with an effect of summer on weight gain identified for the following subgroups: black, Hispanic, and overweight children and adolescents.

**Conclusion:**

There may be a trend in increased rate of weight gain during summer school vacation, particularly for high-risk groups, including certain racial/ethnic populations and overweight children and adolescents. Potential solutions for the problem of accelerated summer weight gain include greater access to recreational facilities, physical activity programming, and summer food programs. Further research in this area is needed as summer weight gain may exacerbate existing health disparities.

## Introduction

One in 3 US children is obese (1) and therefore vulnerable to obesity’s immediate (2,3) and long-term (4) negative health consequences. There has been a marked proliferation of school interventions in recent years (5,6) targeting many elements of the school setting, including the academic curriculum, the physical and social environment, food and physical activity policies, and governance structures (6,7). Qualitative and meta-analytic reviews of this expansive literature suggest that school-based interventions have at least a moderate positive effect on obesity prevention in children (8–11).

Much less attention has been directed toward out-of-school time than in-school time. Yet most students are out of school for 185 to 190 days per year (12). It is important to consider what happens during extended periods of out-of-school time, particularly the summer break, when children and adolescents do not have access to the school lunch program and regular physical activity during recess and physical education. Although an academic “summer achievement slide” has been documented, particularly in groups of people with low socioeconomic status (SES) (13,14), work to date has not considered whether parallel disparities in weight status exist as well.

Several studies (15–21) have examined whether weight gain in children occurs at a faster or more variable rate during summer vacation compared with the school year. Although a recent review considered this body of literature (22), the study was not conducted from a health disparities perspective. The objective of our study was to compile and synthesize research on seasonal patterns of weight gain, with an emphasis on racial/ethnic disparities, to provide greater insight into the role of schools in the prevention of child obesity, the need for summer interventions to control weight gain in children, and the possible need for such programs to focus on certain demographic groups.

## Methods

### Data sources

A systematic search of 2 databases, PubMed and Embase, was used to identify studies for inclusion in this review. Reference lists of identified articles and Google Scholar were also reviewed to identify additional relevant articles. Studies published from January 1, 1990, to August 1, 2013, were considered. Search terms in various combinations were “summer,” “seasonal,” “weight gain,” “BMI,” “school,” “out-of-school time,” and “children.”

### Study selection

Any study that assessed child or adolescent weight before and after the summer vacation was included for review. Study inclusion criteria were the following: 1) a focus on students aged 5 to 17 years; 2) direct assessment of body composition (ie, height and weight and body fat) measured (at a minimum) before and after the summer vacation; 3) English-language articles; and 4) publication in a peer-reviewed journal since January 1, 1990. After exclusions, 7 studies were left for review and qualitative analysis (Figure).

**Figure:**
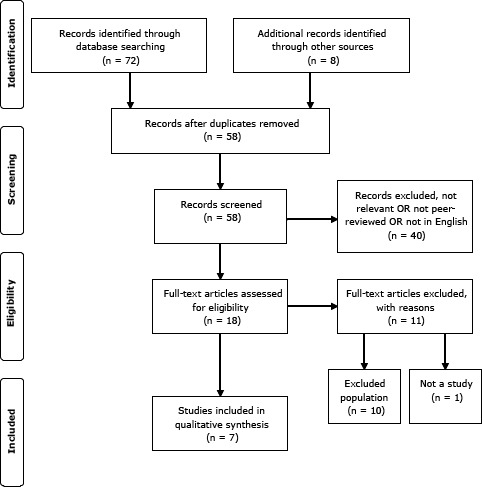
Flow diagram of study identification, screening, eligibility, and inclusion.

To focus on school-time versus out-of-school time (summer vacation) as the mechanism of interest, we excluded studies that examined seasonal growth patterns in children or adolescents outside the context of an academic calendar (ie, from developing countries, where seasonal weather patterns, infectious disease, or growing seasons are the proposed mechanism for differences in rates of weight gain); however, no exclusions were otherwise made on the basis of country of study. Studies published before 1990 were excluded because of the secular changes in growth patterns in children that have taken place since then. Given the small body of literature, we made no exclusions related to the frequency or method of body composition assessment other than the requirement that height and weight, or other measures of body composition, be directly measured rather than self-reported. When available, additional health indicators (eg, blood lipid levels, maximum oxygen consumption) were noted as secondary outcomes. We recorded body mass index (BMI) or percentage-change in BMI when they were reported; those measures are preferred to BMI *z* scores (which are obtained from Centers for Disease Control and Prevention charts that are based on cross-sectional data and may not accurately reflect typical growth patterns in children) when accounting for change in weight status (23,24).

### Data extraction

Data were extracted from the selected studies in the following categories: study purpose, setting, design, population, sample size, data collection method, and findings.

## Results

### Characteristics of studies

The 7 studies selected for review and analysis were published between 2005 and 2013 (Table). Five studies originated in the United States, including 2 studies on American Indian schoolchildren. The other 2 studies took place in Japan and Canada. Participants were from a mix of urban, suburban, and rural settings and represented a range of racial/ethnic groups and income levels. The grade levels of the children who participated in the studies were elementary school (4 studies), middle school (2 studies), and both elementary and middle school (1 study); the mean age ranged from 5 to 12 at baseline. Sample sizes ranged from 17 to 5,380 children, with a total of 10,099 children across all studies. Of the 7 studies, 3 were interventions, 3 were longitudinal observational studies, and 1 study used a repeated cross-section design. Measures of weight gain included objectively-measured BMI (6 studies) and dual x-ray absorptiometry (1 study). The timing and frequency of measurements varied: 5 studies collected measurements at the beginning and end of the school year, and 2 studies collected measurements monthly (all studies used at least 3 time points of measurement). Overall, the studies reported a range of findings, and certain trends occurred by study population.

**Table:** Characteristics of Studies Included (N = 7), Systematic Review on Accelerated Weight Gain Among Children During Summer Versus School Year

Study Purpose	Study Design	Setting	Sample Size, Age Range, Race/Ethnicity, Income Status	Data Collection Method	Major Findings
**Carrel et al, 2007 (** **15** **), United States**					
To determine changes in percentage of body fat, cardiovascular fitness, and insulin levels during the 3-month summer break among overweight children enrolled in a school-based fitness program.	Randomized trial; 1-y lifestyle intervention.	A rural middle school and an academic children’s hospital.	N = 17 9 girls, 8 boys Mean age, 12 y (SD, 0.5 y)	Measurement timing: measurements taken at beginning and end of 1 school year and beginning of next school year. Measurement method: body composition by means of dual x-ray absorptiometry.	While middle-school children were on summer break, mean fitness level decreased; fasting insulin level increased; and percentage body fat increased to levels similar to those seen before the intervention.
**Gillis et al, 2005 (** **16** **), Canada**					
To measure the effect of holidays or season on changes in body weight to determine whether these were reasons for the low success rate of weight-control program participants.	Retrospective, weight-control program.	The Children’s Exercise and Nutrition Centre (Ontario).	N = 73 40 boys, 33 girls Mean age, 10.5 y (SD, 2.8 y) Race/ethnicity: white	Measurement timing: monthly for 1 year. Measurement method: objectively collected height and weight, used to calculate % ideal body weight.	66% of subjects gained weight during the summer months; significant gain in % ideal body weight during July and August compared with other months of the year.
**Kobayashi and Kobayashi, 2006 (** **21** **), Japan**					
To examine whether weight gain during the summer (ie, July through September) is a possible cause of obesity.	Time series.	Elementary schools in Tokyo, Japan.	N = 446 229 girls, 217 boys Age: children in elementary school, grades 1–6 Race/ethnicity: Japanese	Measurement timing: monthly (weight) and 3 times/y (stature) during a 6-y period in 6 cohorts. Measurement method: objectively collected weight and height converted to BMI and degree of obesity.	86% of children exhibited decreased rates of weight gain (using the criterion of “degree of obesity”) during summer compared with the school year. Increased rates of weight gain in summer were observed only among obese children. Children whose weight increased during the summer were observed to spend most of their time indoors.
**Moreno et al, 2013 (** **18** **), United States**					
To examine changes in standardized BMI (BMI *z* score) during school months compared with summer months.	Prospective.	Independent school district in southeast Texas.	N = 3,588 Ages 5–7 y; enrolled in kindergarten at baseline Race/ethnicity: white, black, Hispanic, Asian	Measurement timing: fall and spring of each school year, for 5 y. Measurement method: objectively collected weight and height, used to calculate BMI and BMI *z* score.	There was a significant difference between the school year and summer months with respect to BMI *z* score change, with decreasing BMI *z* score during the school year and increasing BMI *z* score during the summer months; overweight and obese students had significantly greater changes in BMI *z* score than normal-weight students; overweight and obese students decreased BMI *z* score during the school year and increased BMI *z* score during the summer, while normal-weight students increased BMI *z* score during both time periods.
**Smith et al, 2009 (** **19** **), United States**					
To examine whether weight gain during discrete out-of-school periods is occurring and contributing to the prevalence of overweight and obesity among American Indian youth.	Prospective (for subsample relevant for this review).	Wind River Indian Reservation, central Wyoming.	N = 141 Age: children in grades 3, 4, 5, 7 Race/ethnicity: Northern Arapaho and Eastern Shoshone Native American	Measurement timing: beginning of 2 school years and end of 1 summer vacation. Measurement method: objectively collected weight and height, used to calculate BMI.	Significant increases in BMI were found after the summer vacation among numerous age and sex subgroups and among overweight students. Effects were no longer significant, however, when BMI z score was used in place of BMI.
**Von Hippel et al, 2007 (** **17** **), United States**					
To determine whether school or nonschool environments contribute more to childhood overweight (comparing gains in BMI during school vs during summer vacation).	Prospective.	National Center for Education Statistics: the Early Childhood Longitudinal Study.	N = 5,380 Age: children in kindergarten at baseline and in 1st-grade at follow up Race/ethnicity: white, black, Hispanic, other nonwhite	Measurement timing: beginning and end of kindergarten, beginning and end of first grade. Measurement method: objectively collected weight and height, used to calculate BMI.	BMI increase was faster and more variable during the summer than during the school year for kindergarten and 1st grade students. The difference in weight gain between school and summer was especially pronounced for at-risk subgroups (black children, Hispanic children, and children already overweight when entering kindergarten).
**Zhang et al, 2011 (** **20** **), United States**					
To investigate the effects of summer vacation between kindergarten and first grade on growth in height, weight, and BMI for a sample of American Indian children.	Prospective, school-based obesity prevention trial.	Northern Plains Indian Reservation.	N = 454 213 girls, 227 boys Age at baseline: 5.8 y (SD, 0.5 y) Race/ethnicity: American Indian	Measurement timing: beginning and end of kindergarten and grade 1, resulting in 3 intervals: kindergarten school year, summer vacation, 1st-grade school year. Measurement method: objectively collected height and weight.	Although some differences in velocities of weight and height across the 3 intervals were found, no significant differences in velocities of BMI *z* scores were found across intervals controlling for age, sex, intervention condition, socioeconomic status, and weight status at baseline.

Abbreviations: SD, standard deviation; BMI, body mass index.

### General population

Of the 2 studies conducted among the general population in the United States, both identified patterns of accelerated summer weight gain compared with the school year (17,18). In the first study, a large longitudinal study using survey data from the Early Childhood Longitudinal Study — Kindergarten Cohort, BMI measurements at the beginning and end of kindergarten and first grade showed that BMI increase was faster and more variable during the summer than during kindergarten and 1st-grade school years (17). Similarly, in a 5-year longitudinal study conducted in southeast Texas, children generally lost weight during the school year and gained weight during the summer (18).

### Studies by race/ethnicity

Three studies considered populations by race/ethnicity (2 among American Indian children and 1 among Japanese children) (19–21), and 2 studies examined race/ethnicity as a potential moderator (17,18). The remaining 2 studies did not examine race/ethnicity (15,16).

Of the 2 studies of American Indian children, neither identified accelerated summer weight gain compared with the school year (19,20). The first study measured student BMI directly before and after summer vacation in American Indian school children in grades 3 through 7 (19). Significantly higher BMI was noted after summer vacation for numerous age and sex subgroups. These differences were not significant, however, when BMI *z* score was used in place of BMI.

A second study of American Indian children examined growth patterns within a prospective, school-based obesity prevention trial (20). Height and weight were measured on 4 occasions, resulting in 3 intervals: the kindergarten school year, summer vacation, and the 1st-grade school year. After controlling for child age and sex, intervention condition, and weight status at baseline, the study found no significant differences in the velocity of BMI *z* scores across the 3 intervals. The study suggested that weight velocity was lower and height velocity higher during the summer than during the school year. BMI data were not provided (20).

A study of Japanese children (using a time-series design, with weight measured monthly and stature assessed 3 times per year during 6 years) reported that 86% of the children exhibited a decrease in the rate of weight gain (using the criterion of “degree of obesity”) during the summer compared with the school year. This pattern (rate decreases during the summer and rate increases during the school year) is considered typical in Japan according to national surveys on children’s physical growth conducted annually since 1948 (21).

The study that used data from the Early Childhood Longitudinal Study — Kindergarten Cohort, a nationally representative sample, observed negative effects of summer vacation on weight gain that were most pronounced among black and Hispanic children. Moreover, the racial/ethnic gap in weight status increased during summer; no change in this gap was found during the school year (17). In contrast, in a 5-year longitudinal study of students entering kindergarten in southeast Texas, no differences were found in change in BMI *z* score across race/ethnicities (18).

### Overweight and obese children

Two studies considered only overweight/obese students at baseline (15,16); the other 5 studies considered differences according to baseline weight status (17–21). In the study that focused on overweight students participating in a school-based fitness intervention, students’ percentage body fat decreased significantly during the school year. Intervention-associated improvements (in percentage body fat as well as the secondary outcomes of cardiovascular fitness, fasting insulin level, and percentage body fat) that occurred during the school year reversed during the summer, and measurements returned to baseline levels by the beginning of the next school year (15). In the other study of overweight or obese children (16), conducted in the context of a year-long pediatric weight-control program, monthly height and weight measurements showed significant increases in percent ideal body weight during the summer months (July and August) compared with the rest of the year.

Of the 2 studies conducted among American Indian schoolchildren, 1 study observed significant increases in BMI during summer for children at or above the 85th BMI percentile and no changes in BMI for normal-weight students; however, corresponding *z* scores did not change (19). As noted above, the other study among American Indian children found no significant differences in velocity of BMI *z* scores (20). 

Of the 2 studies that considered prospective data from the general population, 1 study observed negative effects of the summer vacation that were most pronounced among children who were classified as overweight when they started kindergarten (17). Similarly, the other study found that overweight and obese students showed increases in BMI *z* score during the summer and decreases during the school year. Normal-weight students showed increases in BMI *z* score during both periods (18).

Finally, in the study conducted among Japanese children, the authors identified differences in rate of weight gain by baseline weight status: the rate of weight gain during summer was greater among overweight or obese children than among their nonoverweight counterparts (21).

## Discussion

Of the 7 studies reviewed, 6 identified accelerated weight gain among school-aged children during the summer vacation compared with the school year for at least a portion of the study population (15–19,21). In the general population, a large nationally representative longitudinal study found evidence of accelerated summer weight gain among kindergarten and 1st-grade children (17), and a 5-year longitudinal study in Texas found evidence of accelerated summer weight gain among elementary students (18).

Findings varied among study populations and by race/ethnicity. Whereas the studies conducted among American Indians (19,20) and in Japan (21) did not observe accelerated summer weight gain compared with the school year for their overall study populations, a large nationally representative longitudinal study in the United States observed pronounced effects of summer vacation on weight gain among black and Hispanic children (17). In contrast, a smaller longitudinal study in Texas did not observe differences by race/ethnicity (18).

We found fairly strong support for accelerated summer weight gain among children who were already overweight or obese; 6 of 7 studies demonstrated this trend across diverse study populations (15–19,21). Both longitudinal studies observed a pronounced effect of summer on weight gain among overweight students (17,18). Similarly, accelerated summer weight gain was observed in 2 studies focusing on overweight and obese children participating in weight-control trials (15,16). In 2 studies, accelerated summer weight gain was limited to children who were overweight at baseline (19,21).

The studies reviewed suggested many potential mechanisms for accelerated summer weight gain. Such mechanisms included decreased physical activity (15,19), increased sedentary behaviors (16–18,21), increased access to unhealthy snacks (17,18,21), unstructured schedules and boredom (15–17), less self-monitoring (15–17), irregular sleep patterns (16,17), and less access to healthier meals through school breakfast and lunch (16,18) during the summer relative to the school year. These mechanisms are purely speculative, however: none of the studies tested these potential mechanisms. Few studies have examined school-year versus summer-time differences in children’s diet, physical activity, and screen-related behaviors.

Overall, findings suggest that school-aged children may gain weight at a faster rate during the summer compared with the school year and that accelerated rates are most pronounced among those at greatest risk of obesity (ie, overweight children and potentially racial/ethnic minority children). Reasons for faster rates of summer weight gain among overweight and racial/ethnic minority children are unclear. The most likely mechanism is SES. Racial/ethnic minority and overweight children are disproportionately represented in low-SES groups. Children from low-SES families may have less access to out-of-school activities such as summer camps (25), which may protect against summer weight gain. Children who attend summer camps engage in greater levels of physical activity during the summer (26), and children who engage in organized activities during the summer are at lower risk for obesity than children who do not engage in organized activities (27).

This pattern parallels the well-documented summer achievement slide in which students’ reading and math scores decline during the summer, particularly in low-SES groups (13,14). As with the summer achievement slide, accelerated summer weight gain among low-SES children may exacerbate disparities. While many interventions have been developed to address declining math and reading skills during the summer (28,29), we are unaware of any programs targeting summer weight gain, particularly among low-SES groups. Although Baranowski and colleagues recently reviewed this body of literature (22), their conclusions focused on differences between overweight and obese children and healthy-weight children and emphasized seasonal differences in physical activity; they did not fully consider the implications by race/ethnicity and health disparities. 

Our review has several implications. Children are engaged in a range of settings during the summer, including the family, community programs, and summer camps. These settings should be considered as possible solutions for unhealthy patterns of weight gain during the summer, when children do not have the regular school-time physical activity opportunities of recess and physical education. Greater access to summer camps and parks and recreation programming, improved food and physical activity policies for summer camps, and increased access to community physical activity resources through the development of joint-use agreements are also potential solutions. Increased use of summer food programs, which provide meals outside of the school year, should also be considered as a potential mechanism for improving eating habits among populations that have unhealthy summer weight gain.

Our study has several limitations. Because of the small number of studies identified, any conclusions drawn from this review are tentative. Relatively broad inclusion criteria were set to identify as many studies as possible. This review did not exclude studies from countries other than the United States. However, cultural or structural differences between countries may prohibit direct comparisons. For example, the summer break in Japan is only approximately 40 days (21), which is shorter than breaks in many school districts in the United States, potentially mitigating any detrimental effects of the summer break on weight gain.

Conclusions from this review are also limited by methodological limitations of the studies identified. Three of the 7 studies reviewed were interventions (15,16,20), and 2 studies were school-based interventions targeting obesity and its behavioral precursors. Although such studies are meaningful and can inform our understanding of summer weight gain, they do not provide a clean test of the association between summer and weight gain. Data from school-based interventions effectively test differences in weight gain between the school year (plus intervention) and the summer (without intervention). This design confounds the absence of school with the absence of an intervention. As noted by von Hippel et al, season also confounds any assessment of differences in weight gain during the school year and summer (17). Other limiting factors include 1) the use of clinical populations (16) which limits the generalizability of findings; 2) the use of data compiled between 1972 and 1998 (for example, Kobayashi and Kobayashi [21]), a period of shifting growth patterns and accelerated weight gain among children worldwide; 3) the absence of studies testing mechanisms of summer weight gain; and 4) differences in the extent to which studies accounted for variations in children’s height and weight that reflect expected growth.

Finally, a limitation in making comparisons across studies is the use of various measures of change in weight status. Although BMI *z* scores may be preferable for comparing weight status on a single occasion, BMI or percentage-change in BMI are preferred for measuring and interpreting changes (23,24). For longitudinal studies of child obesity, change in BMI is recommended as a measure over change in BMI *z* score because *z* scores are derived from charts developed by the Centers for Disease Control and Prevention using cross-sectional data and may not reflect actual growth patterns of children (23). In particular, the study by Moreno et al, which identified no significant differences by race/ethnicity (18), and the study by Zhang et al, which identified no significant differences at all (20), were based on the use of BMI *z* scores.

The studies reviewed suggested that rates of weight gain accelerate during the summer compared with the school year, particularly among racial/ethnic minority and overweight children. Because of the implications for preventing childhood obesity and reducing health disparities, additional research on this topic is needed. To determine whether the findings of the studies in this review reflect a real pattern, additional longitudinal (nonintervention) research is needed to assess in-school versus out-of-school differences in student BMI and differences by race/ethnicity, weight status, and SES. Such research would not only inform clinicians, policy makers, educators, and family members seeking to promote healthy childhood growth but would also help to determine whether summer-time resources should be universally applied or targeted to certain groups to most effectively reduce health disparities.
